# Supplementary material for: Deciphering the Antibacterial Mechanisms of 5-Fluorouracil in Escherichia coli through Biochemical and Transcriptomic Analyses
Source: Antibiotics (Basel). 2024 Jun 5;13(6):528. doi: 10.3390/antibiotics13060528 (PMC11200800; doi:10.3390/antibiotics13060528)
Supplement: Supplementary file 1 [file antibiotics-13-00528-s001.zip › antibiotics-3041910-supplementary.pdf]

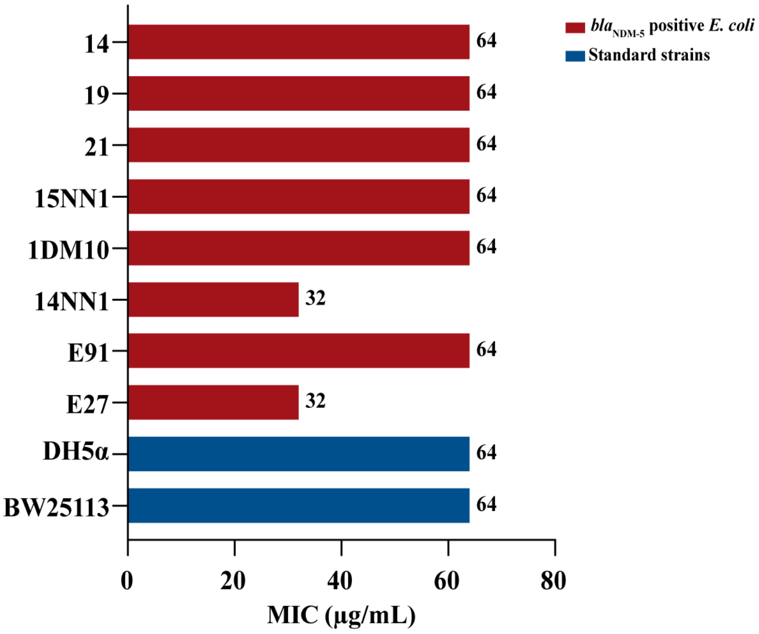

Figure S1 The MIC determination of 5-FU in *E. coli*

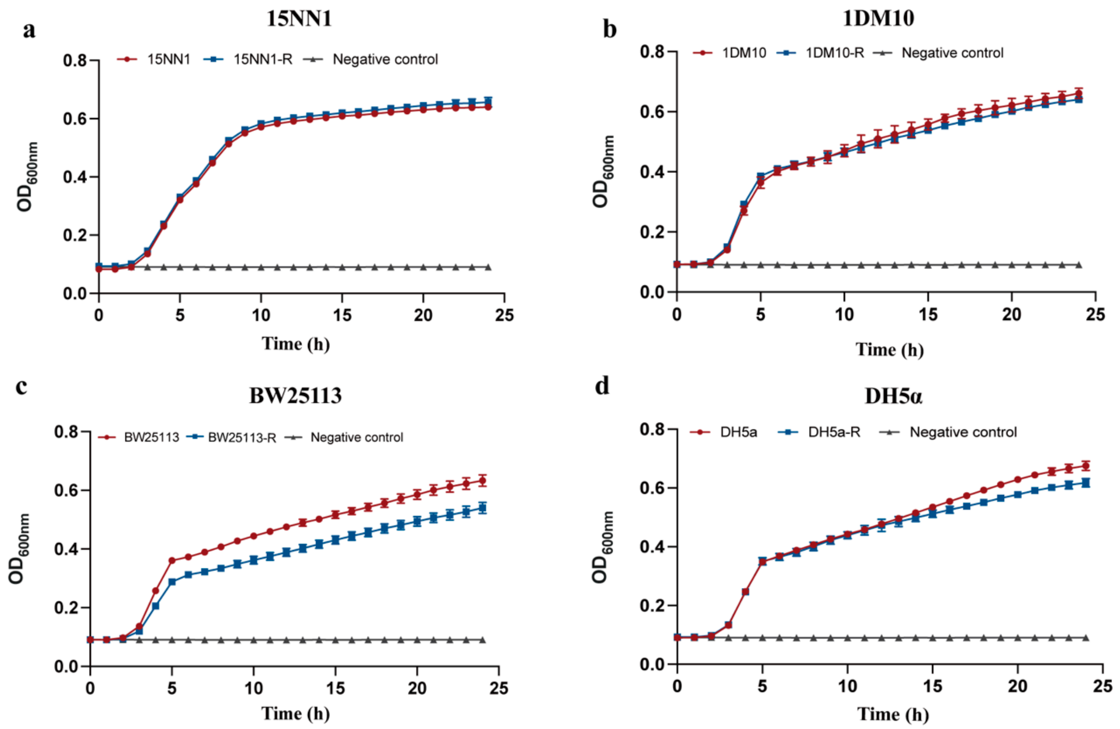

Figure S2 The growth curve of 5-FU-resistant *E. coli* strains. (a) 15NN1; (b) 1DM10; (c) BW25113; (d) DH5α

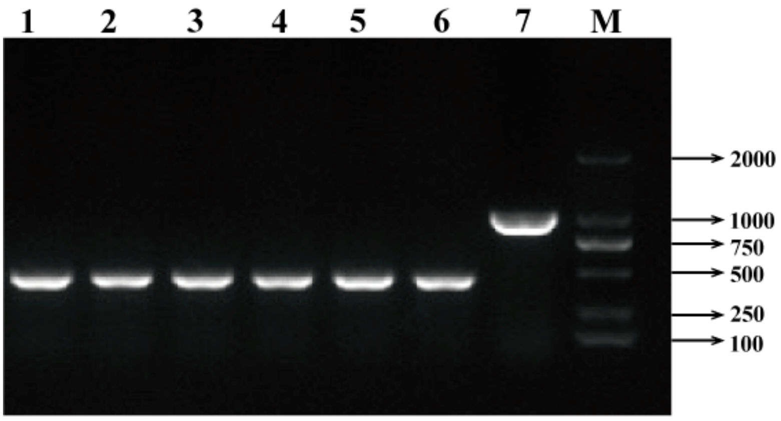

Figure S3 Electrophoresis diagram of the construction identification for *E. coli upp* gene knockout strains. Lanes 1-6 show the knockout strain verification, lane 7 shows the original strain *E. coli* BW25113-IncX3, and M is the marker. The expected agarose gel electrophoresis band size for the original strain is 1011 bp. The knockout strains, which have the chloramphenicol resistance gene removed, show bands smaller than the original strain, indicating successful *upp* gene knockout. The sequences of verification primers were GACGGTTGCACCAAACATGG (Forward) and GCGGCGAAAGAAGACTTGTG (Reverse)

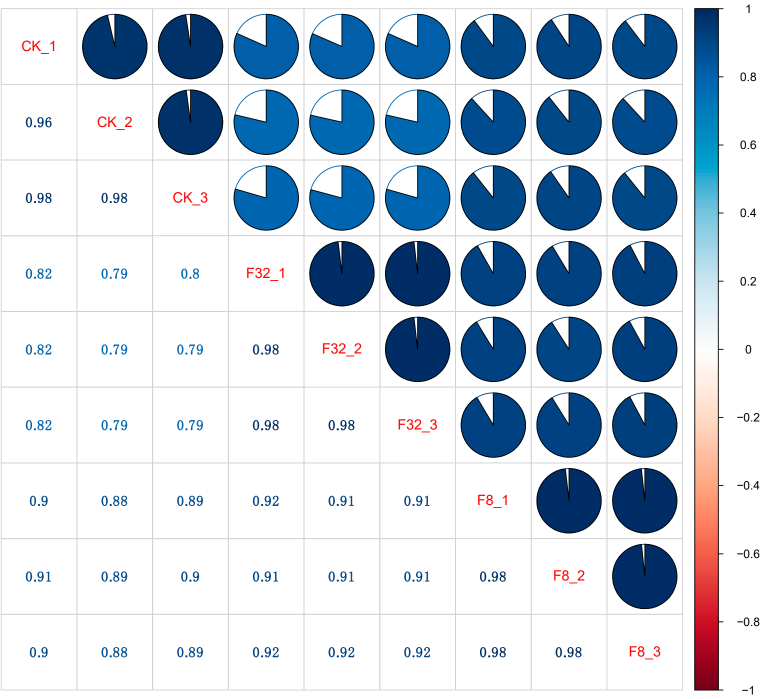

Figure S4 Sample correlation chart

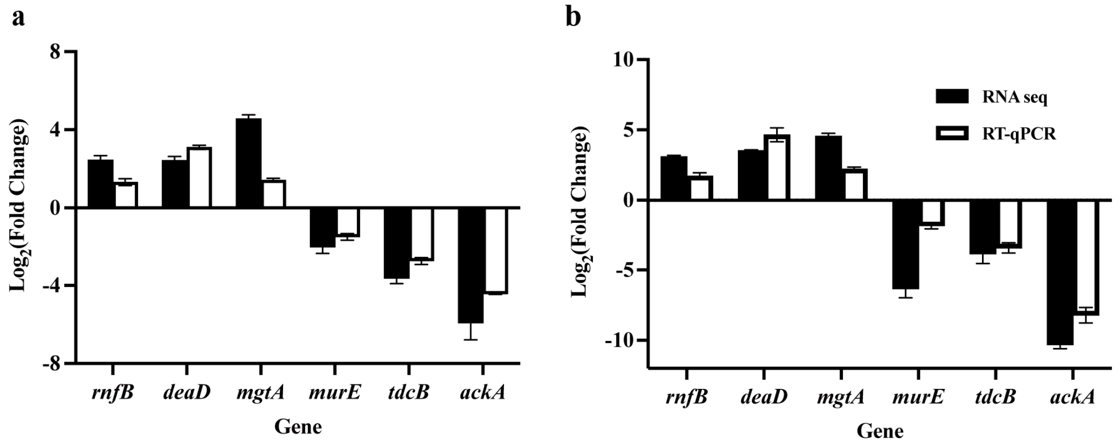

Figure S5 The RT-PCR verification results of six selected DEGs. (a) Treatment with 8 μg/mL 5-FU; (b) Treatment with 32 μg/mL 5-FU

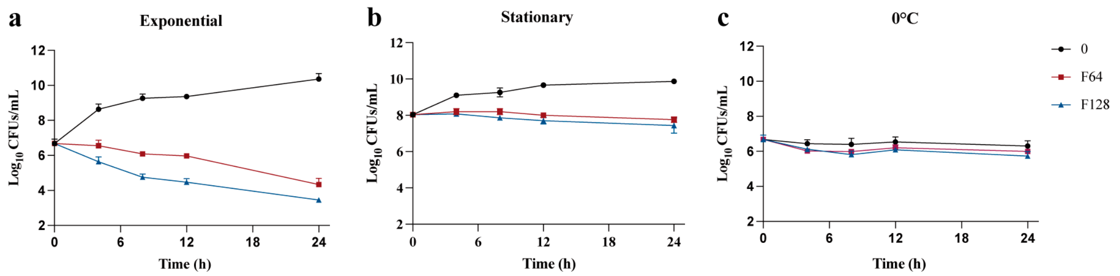

Figure S6 Time-dependent killing curves of *E. coli* 15NN1 treated with 5-FU. (a) Effect of 5-FU against exponential phase *E. coli* 15NN1; (b) Effect of 5-FU against stationary phase *E. coli* 15NN1; (c) Effect of 5-FU against exponential phase *E. coli* 15NN1 at 0°C

Table S1 The MIC of 5-FU against *E. coli*

Strains	MIC (μg/mL)		
	Replicate 1	Replicate 2	Replicate 3
BW25113	64	64	64
BW25113-R	2048	2048	2048
DH5α	64	64	64
DH5α-R	2048	2048	2048
1DM10	64	64	64
1DM10-R	2048	2048	2048
15NN1	64	64	64
15NN1-R	2048	2048	2048
BW25113-IncX3	32	32	32
BW25113-IncX3-△*upp*	1024	1024	1024
BW25113-IncX3-△*upp*-pUC19-*upp*	32	32	32

Table S2 The results of RNA sequencing data quality control

Sample	Raw Reads Number	Filtered Reads Number	Filtered_Q20%	Filtered_GC%	rRNA(%)
Control_1	8517508	8476308	96.96	51.10	0.59%
Control_2	9336740	9293018	96.79	51.49	0.58%
Control_3	11881584	11830650	97.16	51.56	0.62%
F32_1	7438864	7411842	97.83	51.23	1.16%
F32_2	7500748	7477460	97.94	51.08	1.12%
F32_3	12956142	12927556	98.41	51.11	1.15%
F8_1	7950526	7914434	97.08	51.43	1.07%
F8_2	11828456	11774214	96.93	51.12	1.01%
F8_3	13684272	13629472	97.45	51.21	1.07%

Control_1~Control_3 indicates the untreated group, F32_1~ F32_3 represented the 5-FU treatment group of 32 μg/mL, F8_1~ F8_3 represents the 5-FU treatment group of 8 μg/mL

Table S3 Sequence mapping results of the reference genome and sample genome

Sample	Total Reads	Mapped Reads	Unmapped reads	Multiple Mapped Reads	Mapping ratio (%)
Control_1	8476308	8445793	9290	8436503	99.64%
Control_2	9293018	9259563	9259	9250304	99.64%
Control_3	11830650	11791608	10612	11780996	99.67%
F32_1	7411842	7382194	5905	7376289	99.60%
F32_2	7477460	7447550	5213	7442337	99.60%
F32_3	12927556	12870674	7722	12862952	99.56%
F8_1	7914434	7886733	6309	7880424	99.65%
F8_2	11774214	11728294	11728	11716566	99.61%
F8_3	13629472	13580405	12222	13568183	99.64%

Control_1~Control_3 indicates the untreated group, F32_1~ F32_3 represented the 5-FU treatment group of 32 μg/mL, F8_1~ F8_3 represents the 5-FU treatment group of 8 μg/mL

Table S4 The tested bacteria in current study

Strains	Species	Source
14	*Escherichia coli*	animal
19	*Escherichia coli*	animal
21	*Escherichia coli*	animal
15NN1	*Escherichia coli*	animal
14NN1	*Escherichia coli*	animal
1DM10	*Escherichia coli*	animal
E27	*Escherichia coli*	clinical
E91	*Escherichia coli*	clinical
DH5α	*Escherichia coli*	standard strain
BW25113	*Escherichia coli*	standard strain
BW25113-IncX3	*Escherichia coli*	constructed strain

Table S5 Primer sequences of RT-qPCR

Gene		Primer sequence（5'-3'）
16S rRNA	Forward	CCTCAGCACATTGACGTTAC
	Reverse	TTCCTCCAGATCTCTACGCA
rnfB	Forward	TGGCGAAGCTGTGATGCTAA
	Reverse	CCGGACACGCCTGAATACAT
tdcB	Forward	GGTAGTTCGGTGCGTGGTTA
	Reverse	GGTGGCGGTTTAATTGCTGG
murE	Forward	CCACTGGCTGATCTGCTGAA
	Reverse	ACAGCCAAAGAACACCACA
ackA	Forward	GGTCTGACCGAAGTGACCAG
	Reverse	GCGGCATTTTCACCGATACC
mgtA	Forward	GCGGTAGATATTGCCCGTGA
	Reverse	TACCAGCACGCTGAACACAT
deaD	Forward	GCTCTTTCATAAAGCGGCGG
	Reverse	AACTGAGCGGTCTGGTTCTG
recA	Forward	AGGGCGTCACAGATTTCCAG
	Reverse	TTCCGGTAAAACCACGCTGA
